# Insights into Ant Phylogeny from Mitogenomic Sampling Across All Extant Subfamilies

**DOI:** 10.3390/biology15181591

**Published:** 2026-09-09

**Authors:** Nan Song, Yuqiang Xi

**Affiliations:** Henan International Laboratory for Green Pest Control, Henan Engineering Laboratory of Pest Biological Control, College of Plant Protection, Henan Agricultural University, Zhengzhou 450046, China

**Keywords:** Formicidae, mitogenome, gene rearrangement, phylogeny

## Abstract

Ants (Formicidae) represent an ecologically dominant terrestrial lineage, yet resolving their deep phylogenetic relationships requires extensive taxon sampling. In this study, we sequenced four new ant mitochondrial genomes (mitogenomes) and assembled 34 others from public genomic data. Combining these with published sequences yielded a broad dataset of 215 species representing all 17 recognized subfamilies. Three of the four newly sequenced mitogenomes were complete and contained the canonical set of 37 genes, all exhibiting a strong A+T bias. Notably, three species displayed distinct gene rearrangements relative to the ancestral insect mitogenome. Phylogenetic inferences based on nucleotide and amino acid datasets supported the monophyly of Formicidae and all subfamilies represented by multiple species. Under concatenated Maximum Likelihood analyses, *Martialis heureka* (Martialinae) was recovered as the sister group to all other extant ants, with the remaining lineages dividing into poneroid and formicoid clades. Within these groups, key relationships were firmly validated, including Aneuretinae + Dolichoderinae, Heteroponerinae + Ectatomminae, Apomyrminae + Amblyoponinae, and an intriguing Myrmicinae + Formicinae clade that diverges from nuclear phylogenomic topologies. However, extensive sensitivity assessments (including likelihood mapping, alternative partitioning, and Bayesian inference) revealed underlying signal conflict and dataset dependence regarding the exact placements of Martialinae and Leptanillinae. These deep-node uncertainties are driven primarily by substitution saturation and compositional bias. Overall, while mitogenomic data provide powerful resolution for most intersubfamilial relationships, fully resolving the deepest ant divergences will require integrating nuclear genome-scale data under sophisticated models accounting for sequence heterogeneity.

## Simple Summary

Ants (Formicidae) are an ecologically dominant lineage whose deep evolutionary history remains challenging to resolve. To refine their phylogeny, we established a large mitogenomic dataset spanning 215 species across all 17 subfamilies, integrating four newly sequenced mitogenomes with 34 assembled from publicly available genomic data and existing published sequences. Notably, three newly sequenced species exhibited gene rearrangements relative to the ancestral insect mitogenome. Phylogenetic analyses robustly confirmed the monophyly of Formicidae and all subfamilies with multiple representatives. In most analyses, *Martialis heureka* (Martialinae) was recovered as the sister lineage to all other extant ants, which were divided into poneroid and formicoid clades. Crucially, our mitogenomic data supported a sister-group relationship between Myrmicinae and Formicinae, as well as stable relationships such as Aneuretinae + Dolichoderinae, Heteroponerinae + Ectatomminae, and Apomyrminae + Amblyoponinae.

## 1. Introduction

Ants represent one of the most diverse and ecologically dominant lineages of terrestrial organisms worldwide, with over 14,000 formally described extant species [1], a figure that may represent only a third of their actual global richness [2]. As arguably the most evolutionarily successful terrestrial metazoans, they possess unparalleled ecological leverage across virtually all major terrestrial ecosystems [3], accounting for 15–20% of global animal biomass on average [4] and exceeding 25% in the tropics [5]. While their complex social behaviors, foraging strategies, and symbiotic associations [5] make them a premier model system for evolutionary biology [6,7], understanding how this dominance arose relies heavily on resolving their deep evolutionary timeline. Crucially, this biosphere dominance is a relatively recent phenomenon; following their origin in the Late Jurassic to Early Cretaceous [8], ants maintained a modest ecological footprint for their first 60 to 80 million years [4]. This prolonged macroevolutionary lag underscores the critical need for robust phylogenetic frameworks to decipher the ecological and historical factors that ultimately drove the remarkable rise and diversification of these ubiquitous social insects.

All ant species belong to a single family, Formicidae Latreille, 1802. According to AntCat [1], the family comprises 16 extant subfamilies, namely Agroecomyrmecinae, Amblyoponinae, Aneuretinae, Apomyrminae, Dolichoderinae, Dorylinae, Ectatomminae, Formicinae, Leptanillinae, Martialinae, Myrmeciinae, Myrmicinae, Paraponerinae, Ponerinae, Proceratiinae, and Pseudomyrmecinae, alongside an extensive fossil record of 6 extinct subfamilies (Armaniinae, Brownimeciinae, Formiciinae, Haidomyrmecinae, Sphecomyrminae, and Zigrasimeciinae). However, the taxonomic status of three genera (Acanthoponera, Aulacopone, and Heteroponera) remains debated; while some systematists subsume them within Ectatomminae [9], others classify them under a distinct subfamily, Heteroponerinae [10,11,12]. Recognizing Heteroponerinae as a distinct subfamily brings the total number of extant Formicidae subfamilies to 17. Since Brown [13] established the first modern classification of ants, the number and relationships of ant subfamilies have undergone frequent revisions. However, following Bolton [12], subfamilial classification has remained largely stable, with the notable exception of Dorylinae, thanks to major advancements in resolving higher clades via multi-gene and genome-scale phylogenetic analyses. Based on an intuitive integration of extensive morphological evidence, Brown [13] recognized nine ant subfamilies divided into two major lineages: the “myrmecioid complex” (comprising Myrmeciinae, Pseudomyrmecinae, Dolichoderinae, and Formicinae) and the “poneroid complex” (containing Cerapachyinae, Ponerinae, Myrmicinae, Dorylinae, and Leptanillinae). Reflecting contemporary systematic philosophy, his “tentative phylogenetic tree” depicted several subfamilies as paraphyletic, notably nesting Pseudomyrmecinae within Myrmeciinae and Myrmicinae within Ponerinae [3].

Using a seven-gene nuclear dataset from 162 taxa, Brady et al. [14] estimated divergence dates for the major ant clades and divided Formicidae into three primary lineages: the leptanilloid, poneroid, and formicoid clades. Analyzing 4.5 kb of sequence data from six genes across 139 genera, Moreau et al. [15] recovered a largely congruent, well-resolved phylogeny. Both studies placed the subfamily Leptanillinae (the leptanilloid clade) as the sister group to all other extant ants. However, the discovery of *Martialis heureka* introduced an alternative hypothesis: that *M. heureka* (Martialinae) is instead the sister lineage to all remaining ants [16]. Most recently, a phylogenomic analysis by Tao et al. [17] found Martialinae to be the sister group to all ants except Leptanillinae, adding another layer to this foundational phylogenetic question.

In this study, we sequenced four new ant mitochondrial genomes (mitogenomes) and assembled an additional 34 from publicly available genomic data. Integrating these with previously published sequences yielded a broad dataset of 227 species. This includes 215 ant species representing all 17 currently recognized ant subfamilies, along with 12 hymenopteran outgroup species. This extensive mitogenomic sampling allows us to rigorously test existing hypotheses of subfamilial relationships within Formicidae and provide deeper insights into the evolutionary history of the group.

## 2. Materials and Methods

### 2.1. Taxon Sampling

Ant specimens representing four species (*Camponotus consobrinus*, *Iridomyrmex anceps*, *Polyrhachis argentosa*, and *Solenopsis* sp.) were collected from the University of the Sunshine Coast (UniSC, Sippy Downs; 26°42′58″ S, 153°03′34″ E) in 2023. Sampling did not require any specific permits or approvals. Specimens were preserved in absolute ethanol at −20 °C prior to DNA extraction. All newly sampled voucher specimens (AU_W4: *C. consobrinus*, AU_W10: *Solenopsis* sp., AU_W11: *I. anceps*, AU_W13: *P. argentosa*) are deposited at the Henan International Laboratory for Green Pest Control, College of Plant Protection, Henan Agricultural University.

Species identification was performed using an integrated approach combining adult morphology and molecular analysis. Specimens were examined under a Leica M205 A stereomicroscope (Leica Microsystems, Wetzlar, Germany) and identified based on key diagnostic characters (e.g., mouthpart, propodeum and antennal morphology) following published taxonomic keys [18,19]. For molecular validation, mitochondrial *cox1* gene fragments were sequenced and queried against the Barcode of Life Data System (BOLD) database (BOLD: https://v4.boldsystems.org/, accessed on 1 August 2026) [20]. Species-level designations were confirmed only for sequence matches yielding a sequence identity ≥ 98%.

### 2.2. Mitogenome Sequencing, Assembly and Annotation

Genomic DNA was isolated from the thoracic muscle tissue of a single specimen preserved in absolute ethanol using the TIANamp Genomic DNA Kit (Tiangen Biotech Co., Ltd., Beijing, China), following the manufacturer’s protocol. DNA concentration and quality were quantified using a Q5000 nucleic acid protein analyzer (Quawell Technology, Inc., San Jose, CA, USA).

Genomic DNA libraries with a target insert size of 400 bp were constructed using the TruSeq Nano DNA High Throughput Library Prep Kit (Illumina, Inc., San Diego, CA, USA) following the manufacturer’s instructions. High-throughput sequencing was performed on the DNBSEQ-T7RS platform (Personal Biotechnology Co., Ltd., Shanghai, China) using a 150-bp paired-end strategy, generating a minimum of 20 Gb of raw data per sample. Initial quality assessment was performed using Fastp v0.23 [21]. Adapter sequences were removed and low-quality reads were filtered using Trimmomatic v0.32 [22]. Only reads with a Q30 score of at least 97.53% were retained for subsequent de novo assembly.

Mitogenomes were assembled de novo from the high-quality reads using GetOrganelle v1.7.7.1 [23] with the “animal mitochondrial” preset and employing the GetOrganelleDB 0.0.1 reference database. To assess whether the mitogenome was complete and circular, GetOrganelle’s automated graph-based validation workflow was used, employing an iterative read extension process with K-mer sizes ranging from 21 to 85. Circularity was algorithmically verified by resolving terminal repeats within the assembly graph into a single, closed-loop path. Assembly integrity was further confirmed by identifying all 37 typical metazoan mitochondrial genes. Additionally, MitoZ v3.6 [24] was used to identify and assemble target-associated reads. These results were cross-checked with the GetOrganelle assemblies to supplement and yield more complete final sequences. We calculated nucleotide coverage statistics using Geneious R11 [25] by mapping high-quality sequencing reads back to the assembled mitogenome. For the 34 additional mitogenomes reconstructed from published genomic data [11], assemblies were performed using either GetOrganelle v1.7.7.1 [23] or MitoZ v3.6 [24].

Initial de novo gene annotation was executed using MITOS v2 [26]. Protein-coding gene (PCG) and ribosomal RNA (rRNA) boundaries were manually refined using multiple sequence alignments with closely related mitogenomes to guarantee accuracy. Secondary structures of transfer RNAs (tRNAs) were predicted using MITOS2 [26] and subsequently verified using tRNAscan-SE (https://trna.ucsc.edu/tRNAscan-SE/, accessed on 1 August 2026) [27] (“Invertebrate Mito” genetic code, score cutoff = 10.0). To distinguish true structural variations—such as missing stems or arms—from prediction or annotation artifacts, all predicted structures were manually inspected, aligned, and cross-verified against homologous tRNA sequences from closely related ant species. All newly sequenced mitogenomes are available in GenBank format as Supplementary Materials. Gene extraction, base composition calculations, and gene order visualization were conducted using the PhyloSuite v2 [28,29]. Pairwise gene order comparisons and rearrangement event analyses were performed using CREx v2 [30] for the three newly sequenced mitogenomes (i.e., *P. argentosa*, *I. anceps*, and *Solenopsis* sp.), each containing the full set of 37 mitochondrial genes, using *Drosophila melanogaster* as the ancestral insect reference.

### 2.3. Phylogenetic Analyses

The 13 PCGs were aligned using TranslatorX v1.1 [31] to preserve the open reading frame according to the invertebrate mitochondrial genetic code, followed by trimming manually. All individual gene alignments were concatenated using PhyloSuite v2 [28,29]. To evaluate the phylogenetic signal and potential biases, three distinct datasets were compiled: (1) PCG_NT123: Nucleotide sequences of the 13 PCGs including all three codon positions; (2) PCG_NT12: Nucleotide sequences of the 13 PCGs including the first and the second codon positions; and (3) PCG_AA: Amino acid sequences of the 13 PCGs. Summary statistics for each alignment were calculated using AMAS v1.0 [32] (Table S1). Substitution saturation of the nucleotide datasets was evaluated using DAMBE v7.3.1 [33]. In addition, sequence compositional heterogeneity was assessed using AliGROOVE v1.06 [34].

The species included in the phylogenetic analysis are listed in Table S2. The dataset includes 215 ingroup species across 17 subfamilies of Formicidae, following the classification of Romiguier et al. [11] (detailed species and genus counts are provided in Table S3), and 12 outgroup species from five families: Vanhorniidae (1), Vespidae (4), Bethylidae (2), Chrysididae (3), and Dryinidae (2). Phylogenetic reconstructions were performed using Maximum Likelihood (ML) and Bayesian Inference (BI) criteria.

ML analyses were executed in IQ-TREE v2.0 [35]. Gene-specific partitioning of the datasets was performed, with optimal substitution models and partitioning schemes determined using ModelFinder [36]. Two partitioning strategies were employed: one based on genes only, and another based on gene × codon-position combinations. Both analyses were conducted under an edge-proportional partition model. Nodal support was assessed using 10,000 ultrafast bootstrap (UFBoot) replicates [37] alongside 10,000 SH-aLRT tests [38]. Nodes were considered strongly supported if they received both UFBoot support ≥ 95% and SH-aLRT support ≥ 80%.

BI analysis was performed exclusively on the amino acid dataset (PCG_AA) to optimize computational efficiency, using PhyloBayes MPI v1.8 [39]. For this dataset, we implemented the site-heterogeneous CAT-GTR mixture model [40] combined with the empirical mtART exchangeability matrix, discrete Gamma-distributed site rates, and a Birth–Death prior (CAT+GTR+G+BD). Convergence of MCMC chains was assessed using Tracer v1.7.2 [41]. After discarding initial burn-in samples (20%), majority-rule consensus trees and Bayesian posterior probabilities (BPP) were computed from the remaining iterations. Due to computational resource constraints, a single MCMC chain was executed. Clades with BPP ≥ 0.9 were considered strongly supported.

Alternative tree topologies were evaluated using Four-cluster Likelihood Mapping (FcLM, [42]) as implemented in IQ-TREE v2.0 [35], applied separately to the three datasets. This approach evaluates phylogenetic signal and unresolved noise among four predefined lineages by mapping the likelihoods of three alternative unrooted topologies for randomly sampled quartets onto a ternary diagram. For the amino acid dataset (PCG_AA), the PMSF model (LG+C20) was employed, whereas the GTR+F+I+R9 and GTR+F+I+R10 models were used for the PCG_NT12 and PCG_NT123 datasets, respectively.

## 3. Results

### 3.1. Genome Sequencing and Assembly

Whole-genome sequencing yielded total output ranging from 22.36 Gb (22,361,483,700 bp) in *P. argentosa* to 31.02 Gb (31,015,378,500 bp) in *C. consobrinus*. Complete assembled mitogenome lengths ranged from 15,720 bp to 17,321 bp (*C. consobrinus*: 17,321 bp; *I. anceps*: 17,077 bp; *P. argentosa*: 16,242 bp; *Solenopsis* sp.: 15,720 bp). GetOrganelle successfully assembled *Solenopsis* sp., *I. anceps*, and *P. argentosa* into single circular scaffolds, all of which were independently verified by MitoZ. For *C. consobrinus*, GetOrganelle de novo assembly yielded five non-overlapping scaffolds: Scaffold 1 (2052 bp, encoding *trnL2*, *cox2*, *trnQ*, *trnK*, *trnD*, *atp8*, -*trnE*, and *atp6*), Scaffold 2 (3063 bp, encoding *nad2*, *trnW*, -*trnC*, -*trnY*, and *cox1*), Scaffold 3 (1777 bp, encoding *nad6* and *cob*), Scaffold 4 (5481 bp, encoding *cox3*, *trnG*, *nad3*, *trnA*, *trnR*, *trnN*, *trnS1*, *trnE*, -*trnF*, -*nad5*, -*trnH*, -*nad4*, -*nad4l*, and *trnT*), and Scaffold 5 (4948 bp, encoding *rrnS*, *rrnL*, *trnL1*, and *nad1*). Five tRNA genes (*trnI*, *trnM*, *trnP*, *trnS2*, and *trnV*) were not recovered. In comparison, MitoZ recovered only a 4490 bp fragment encoding *nad3*, *trnA*, *trnR*, *trnN*, *trnS1*, *trnE*, -*trnF*, -*nad5*, -*trnH*, -*nad4*, -*nad4l*, and *trnT*. This sequence corresponded to a partial segment of Scaffold 4, as assembled by GetOrganelle. Despite partial assembly fragmentation, all 13 protein-coding genes (PCGs) were recovered at full length without internal gaps, supporting the inclusion of *C. consobrinus* in downstream phylogenetic analyses.

Mean sequencing depth was high across samples: 3580.2× for *Solenopsis* sp. (range: 1–7197×), 6661.8× for *I. anceps* (range: 1–12,214×), and 20,074.2× for *P. argentosa* (range: 1–34,446×). These high sequencing depths reflect a strong abundance of mitochondrial reads, supporting high-confidence genome assemblies.

### 3.2. Mitogenome Organization and Composition

**Figure 1:**
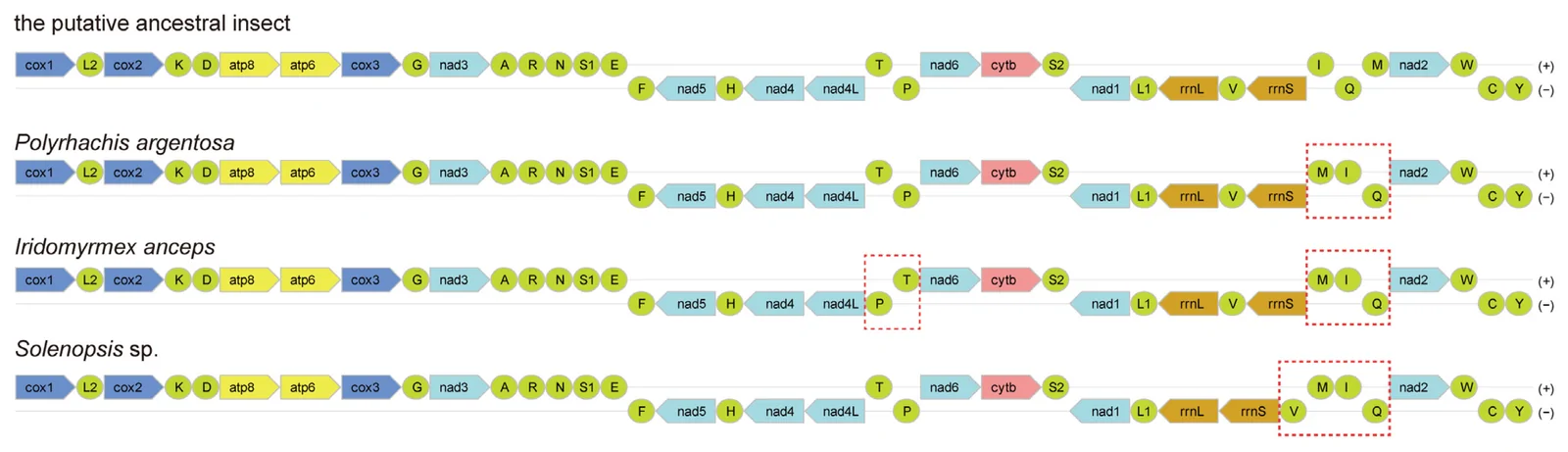
Comparison of mitochondrial genome structures among *Polyrhachis argentosa*, *Iridomyrmex anceps*, *Solenopsis* sp., and the putative ancestral insect. Gene rearrangements are indicated by red dashed lines.

Due to the incomplete assembly and missing genes in *C. consobrinus*, structural and compositional analyses were restricted to the remaining three species. Genome annotation identified the full set of 37 canonical mitochondrial genes: 13 PCGs, 22 transfer RNA genes (tRNAs), and two ribosomal RNA genes (rRNAs) in *Solenopsis* sp., *P. argentosa*, and *I. anceps* (Figure 1). All three assembled mitogenomes fell within the typical insect length range of 15–18 kb [43]. The three new mitogenomes exhibited a strong A+T bias, with overall A+T content ranging from 80% (*P. argentosa*) to 81.3% (*I. anceps*). The major strand exhibited a negative GC-skew, with values ranging from −0.41 (*P. argentosa*) to −0.368 (*Solenopsis* sp.).

**Figure 2:**
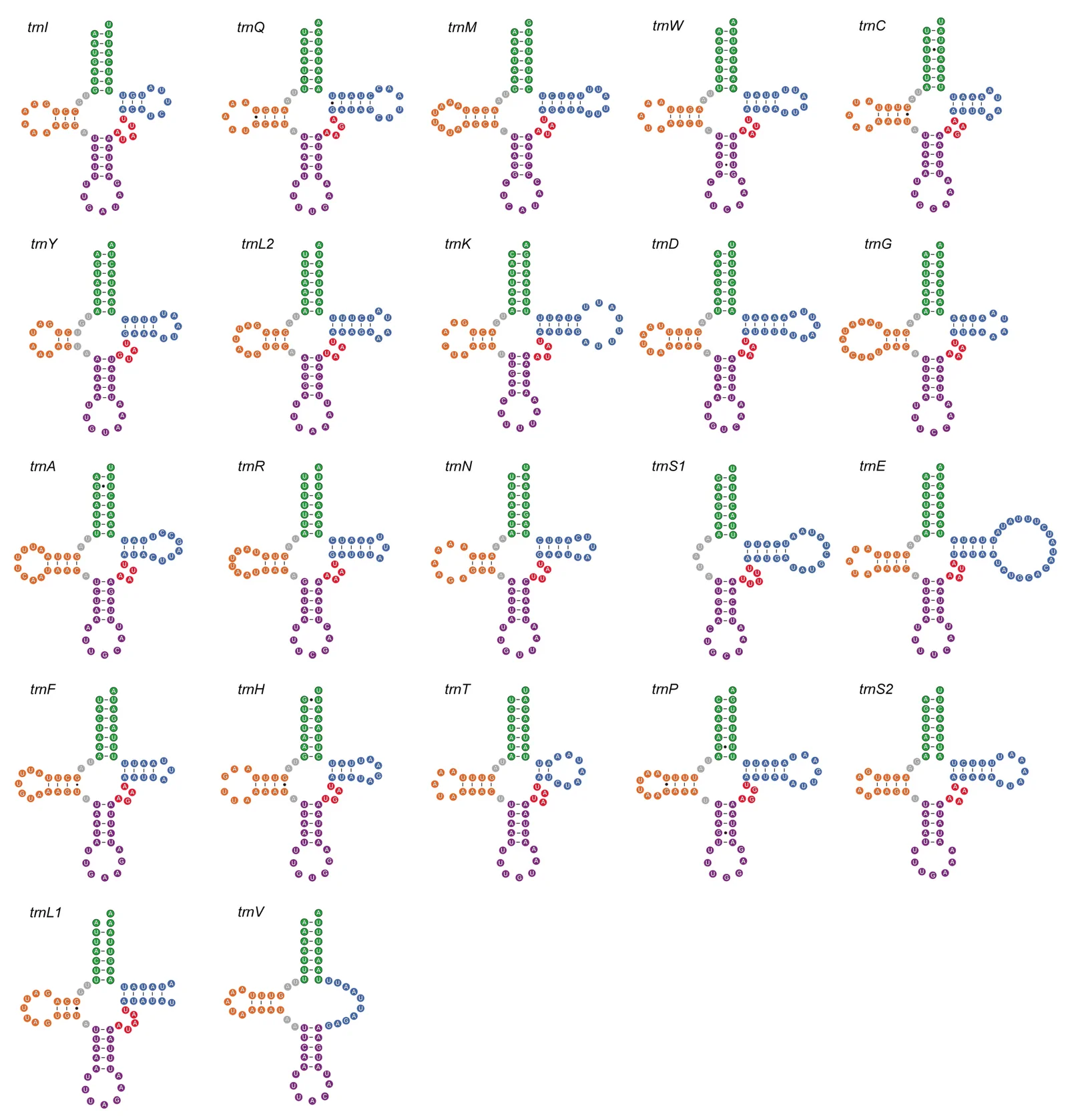
Predicted secondary structures of the 22 mitochondrial tRNAs in *Polyrhachis argentosa*. Functional domains are color-coded: green (amino-acyl arm), orange (dihydrouracil arm), purple (anticodon arm), red (variable arm), and blue (TψC arm). Watson–Crick pairings are indicated by bars; G-U wobble pairs are indicated by dots.

Individual tRNA lengths ranged from 53 bp (*trnR* in *Solenopsis* sp.) to 80 bp (*trnE* in *P. argentosa*). While most tRNAs folded into the typical cloverleaf secondary structure, atypical features were observed in several genes: *trnS1* lacked the DHU stem across three species, *trnR* lacked the acceptor stem in *Solenopsis* sp., and *trnV* lacked the TψC in *P. argentosa* (Figure 2). Both rRNA genes, *rrnS* and *rrnL*, are encoded on the minor strand. The lengths of *rrnS* range from 717 bp (*P. argentosa*) to 793 bp (*I. anceps*), while *rrnL* ranges from 1144 bp (*Solenopsis* sp.) to 1368 bp (*I. anceps*).

Compared to the putative ancestral insect mitogenome [43], novel gene rearrangements were detected in three species (Figure 1), including the inverted *trnM-trnI-trnQ* (vs. ancestral *trnI-trnM-trnQ*) in *P. argentosa*, the translocation of *trnP-trnT* (vs. ancestral *trnT-trnP*) alongside *trnM-trnI-trnQ* (vs. ancestral *trnI-trnM-trnQ*) in *I. anceps*, and the translocation of *trnV* and inverted rearrangement of *trnM-trnI-trnQ* (vs. ancestral *trnI-trnM-trnQ*) in *Solenopsis* sp. CREx analysis revealed that transposition was the primary mechanism underlying the gene rearrangements observed in the three newly sequenced mitogenomes. Comparative analysis of gene orders across 193 relatively complete mitogenomes (containing 34–37 mitochondrial genes) revealed that 16 species exhibit the *trnP-trnT* rearrangement. Among these, 14 species belong to *Pheidole* (Myrmicinae), one to *I. anceps* (Dolichoderinae), and one to the outgroup species *Parischnogaster mellyi* (Vespidae, Stenogastrinae). Additionally, the *trnM-trnI-trnQ* gene arrangement was identified in 110 species; however, these species are dispersed across nine subfamilies (Myrmicinae, Pseudomyrmecinae, Dolichoderinae, Ponerinae, Proceratiinae, Formicinae, Paraponerinae, Dorylinae and Amblyoponinae).

### 3.3. Phylogenetic Inference

**Figure 3:**
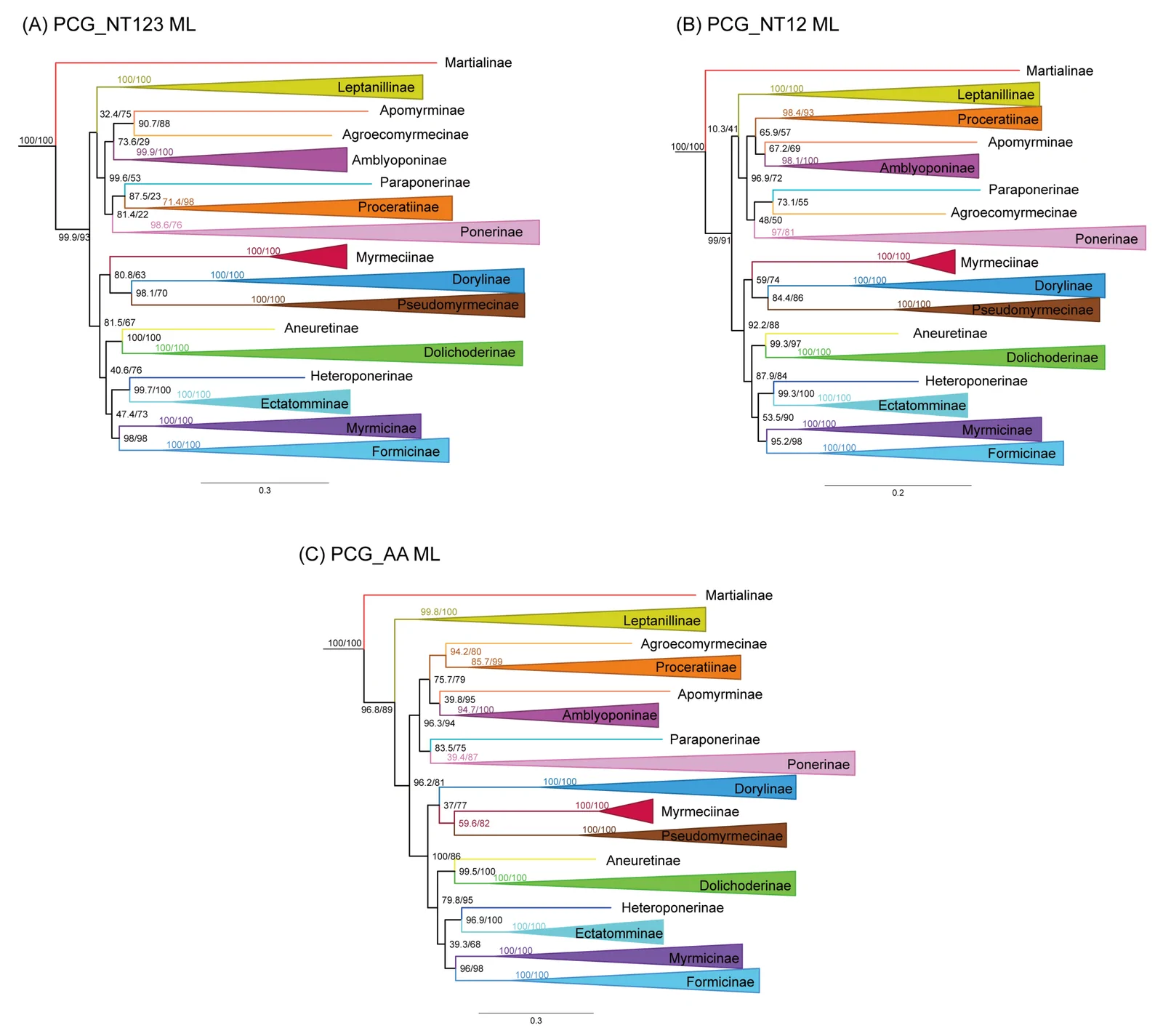
Phylogenetic relationships inferred from maximum likelihood analyses of three gene-partitioned datasets: (**A**) PCG_NT123, (**B**) PCG_NT12, and (**C**) PCG_AA. Major lineages are collapsed for clarity, with triangle lengths corresponding to the longest terminal branch in each collapsed clade. Node labels indicate SH-aLRT support/UFBoot values (%). Clades with UFBoot support < 95% and/or SH-aLRT support < 80% are considered unresolved ambiguous clades. The scale bar indicates substitutions per site. The full trees are provided in Supplementary Figures S1–S3.

All analyses recovered Formicidae as a monophyletic group (SH-aLRT = 100%, UFBoot = 100%, BPP = 1.00, Figure 3 and Figures S1–S4). Within Formicidae, all subfamilies represented by multiple species were also recovered as monophyletic: Leptanillinae, Proceratiinae, Amblyoponinae, Ponerinae, Myrmeciinae, Dorylinae, Pseudomyrmecinae, Dolichoderinae, Ectatomminae, Myrmicinae, and Formicinae.

Regarding subfamilial relationships, *Martialis heureka* (Martialinae) was supported as the sister lineage to all other Formicidae in ML analyses under gene partition schemes. ML analysis based on the amino acid dataset positioned Leptanillinae as the second diverging clade, sister to the remaining Formicidae. Although this arrangement received strong SH-aLRT support (96.8%), the UFBoot support was below the threshold for strong confidence (89%). In contrast, ML analyses based on nucleotide datasets (PCG_NT123 and PCG_NT12) grouped Leptanillinae with a clade comprising Paraponerinae, Apomyrminae, Agroecomyrmecinae, Proceratiinae, Amblyoponinae, and Ponerinae. However, this placement was supported by neither SH-aLRT nor UFBoot.

Overall, excluding Martialinae, ML analyses divided Formicidae into two major clades. The first major clade comprised Paraponerinae, Apomyrminae, Agroecomyrmecinae, Proceratiinae, Amblyoponinae, and Ponerinae, with or without Leptanillinae (included in nucleotide analyses but excluded in amino acid analyses). The second major clade contained Dorylinae, Myrmeciinae, Pseudomyrmecinae, Aneuretinae, Dolichoderinae, Heteroponerinae, Ectatomminae, Myrmicinae, and Formicinae. Although dataset types shared identical taxon compositions within these two major clades, inter-subfamilial relationships varied across analyses. Nevertheless, several sister-group relationships were recovered: Aneuretinae + Dolichoderinae, Heteroponerinae + Ectatomminae, and Myrmicinae + Formicinae.

For the BI analyses, evaluation of the MCMC trace files in Tracer v1.7.2 indicated incomplete convergence for the PhyloBayes runs. Although divergence time and topology estimations yielded acceptable effective sample sizes (ESS = 375 and 680, respectively), other model parameters exhibited low values (ESS < 200). Consequently, the BI results were treated as reference data to complement and validate the ML analyses. The BI topology based on the amino acid dataset was largely congruent with the corresponding ML tree, particularly regarding the basal placements of Martialinae and Leptanillinae (Figure S4). Furthermore, several key sister-group relationships received strong support in the BI analysis, including Apomyrminae + Amblyoponinae (BPP = 0.91), Heteroponerinae + Ectatomminae (BPP = 1.00), Dolichoderinae + Aneuretinae (BPP = 1.00), and Myrmicinae + Formicinae (BPP = 1.00).

**Figure 4:**
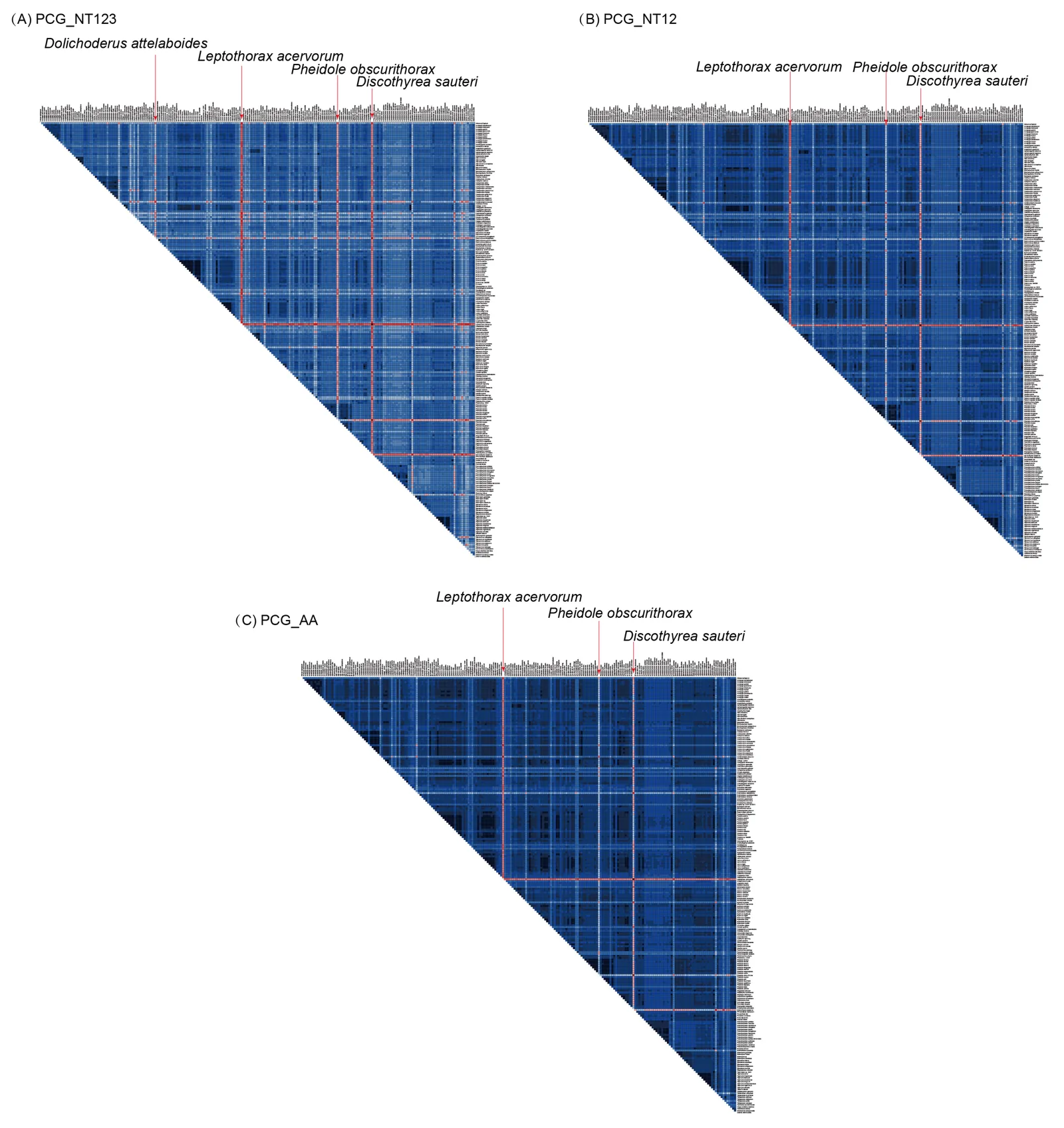
AliGROOVE heat maps of pairwise sequence comparisons for datasets (**A**) PCG_NT123, (**B**) PCG_NT12, and (**C**) PCG_AA. Mean sequence similarity scores range from −1 (high sequence heterogeneity, indicated in red) to +1 (rate similarity across all comparisons). The color gradient ranges from dark blue (high sequence similarity, score +1) to red (high sequence heterogeneity, score −1). Taxa exhibiting marked sequence heterogeneity are highlighted with larger font sizes and red arrows.

Substitution saturation analysis indicated that first and second codon positions across both nucleotide datasets (PCG_NT12 and PCG_NT123) were unsaturated. Although the third codon positions yielded index of substitution saturation (*Iss*) values lower than the critical value under a symmetrical tree topology (*Iss*.*cSym*), *Iss* exceeded *Iss*.*cAsym* when the number of taxa evaluated (*N*_OTU_) exceeded 4, suggesting substantial substitution saturation at the third codon position (Table S4). Compositional heterogeneity tests revealed significant non-stationarity in four species (*Dolichoderus attelaboides*, *Leptothorax acervorum*, *Pheidole obscurithorax*, and *Discothyrea sauteri*) for dataset PCG_NT123 and in three species (*L. acervorum*, *P. obscurithorax*, and *D. sauteri*) for datasets PCG_NT12 and PCG_AA (Figure 4). Nonetheless, the overall compositional heterogeneity across all three complete datasets was minor. To ensure comparability across analytical conditions, we removed the union of these heterogeneous taxa (all four species) from all matrices, yielding a uniform reduced dataset of 223 taxa. Reanalysis of these reduced datasets using the same model settings as the preliminary ML analysis yielded placements for major basal ant lineages largely congruent with those inferred from the full 227-taxon datasets (Figures S5–S7). Conversely, evaluating a gene × codon-position partitioning scheme on the 227-taxon nucleotide datasets (PCG_NT123 and PCG_NT12, Figures S8 and S9) produced topologies that differed substantially from those generated under gene-based partitioning. Although model-selection comparisons indicated that the gene × codon-position scheme was statistically preferred (Table S5), this better-fitting model unexpectedly yielded anomalous topologies. Notably, both gene × codon-position analyses nested *M. heureka* deeply within the family, though without significant statistical support, and reconstructed *Paraponera clavata* (Paraponerinae) in the most basal position.

### 3.4. Four-Cluster Likelihood Mapping Analysis

**Figure 5:**
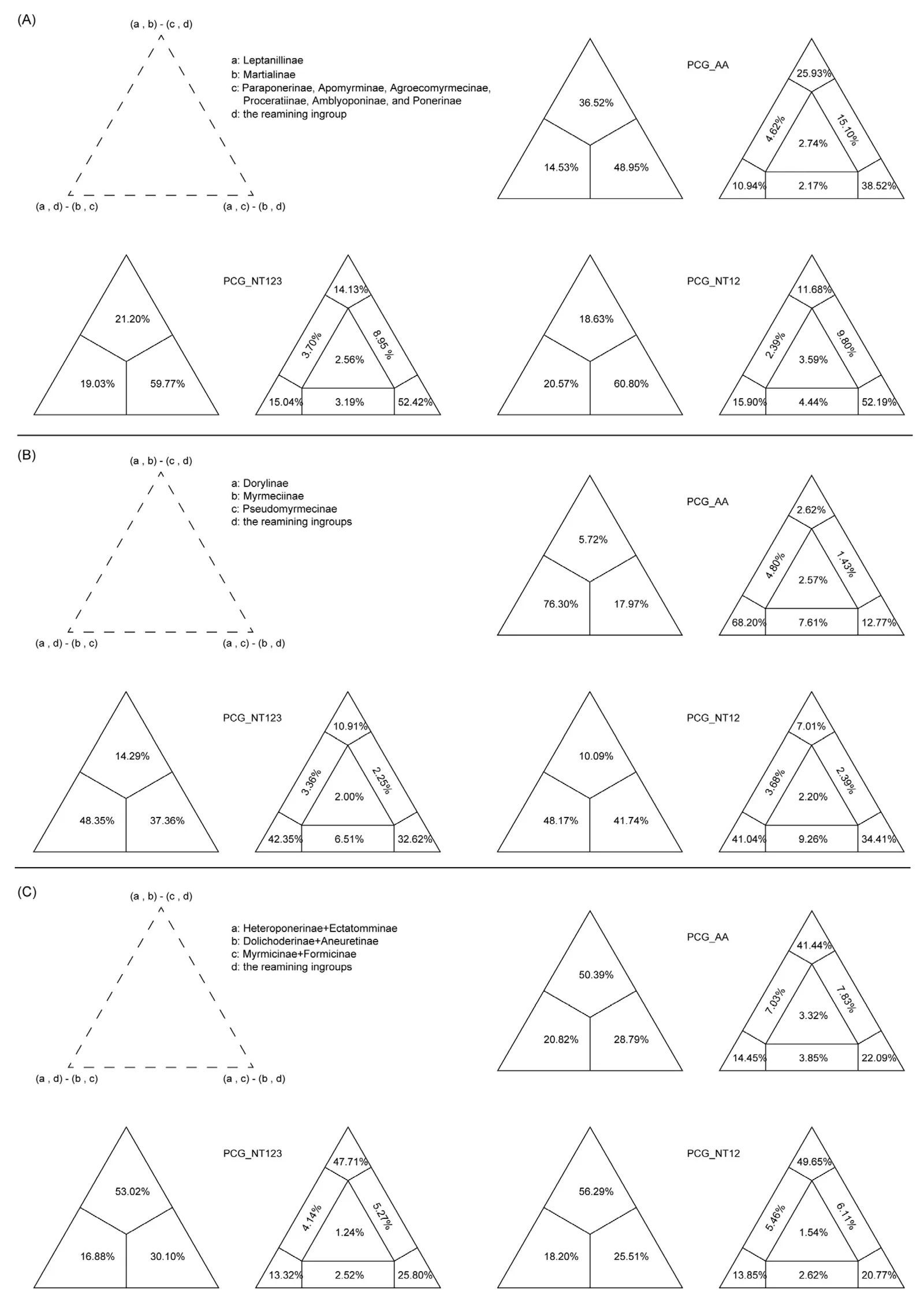
Four-cluster likelihood mapping (FcLM) analyses evaluating phylogenetic signal across three datasets (PCG_NT123, PCG_NT12, and PCG_AA). (**A**) Phylogenetic placement of Leptanillinae relative to other ant lineages. (**B**) Inferred relationships among Dorylinae, Myrmeciinae, and Pseudomyrmecinae. (**C**) Relationships among (Heteroponerinae + Ectatomminae), (Dolichoderinae + Aneuretinae), and (Myrmicinae + Formicinae). Values in each triangle represent the percentage of quartets supporting the respective topologies.

Regarding the phylogenetic placement of Leptanillinae, FcLM analysis (Figure 5) revealed that nucleotide datasets provided strong phylogenetic signal (≥59.77%) placing Leptanillinae as sister to a clade comprising Paraponerinae, Apomyrminae, Agroecomyrmecinae, Proceratiinae, Amblyoponinae, and Ponerinae. Similarly, the amino acid dataset showed more support for a Leptanillinae + (Paraponerinae + Apomyrminae + Agroecomyrmecinae + Proceratiinae + Amblyoponinae + Ponerinae) clade (48.95%), though there was some support for a Leptanillinae + Martialinae relationship (36.52%).

For the relationships among Dorylinae, Myrmeciinae, and Pseudomyrmecinae, all three datasets predominantly supported a sister-group relationship between Myrmeciinae and Pseudomyrmecinae, although a notable proportion of signal (≥48.17%) supported an alternative sister relationship between Dorylinae and Pseudomyrmecinae. Finally, regarding the subfamily groups, across all three datasets there was strong support (≥50.39%) for a sister-group relationship between (Heteroponerinae + Ectatomminae) and (Dolichoderinae + Aneuretinae).

## 4. Discussion

Mitogenomes have been widely employed as molecular markers in insect phylogenetics. Compared to nuclear genome-scale data, mitogenomes offer distinct advantages, including lower sequencing costs and straightforward assembly—largely due to their high cellular copy numbers (typically 1000–10,000 copies per cell; [44,45]). Furthermore, their structural stability simplifies gene annotation. Despite these benefits, mitogenomic studies focusing on ant phylogeny remain surprisingly limited. In this study, we reconstructed the phylogenetic relationships within Formicidae by combining newly sequenced mitogenomes, mitogenomes extracted from existing genomic datasets, and previously published data. Our analyses robustly support both the monophyly of Formicidae and that of its major species-rich subfamilies. Additionally, our findings clarify several inter-subfamily relationships that were either disputed or unresolved in previous studies. Overall, these results demonstrate the utility and effectiveness of mitogenomes in resolving ant phylogenetic relationships.

### 4.1. Deep Diversification and Conflicting Signals Among Early-Branching Ant Lineages

Resolving the deepest node in ant phylogeny remains one of the most challenging problems in systematic entomology. Early molecular studies relying on limited gene fragments frequently recovered Leptanillinae as sister to all remaining Formicidae [14,15]. This hypothesis was challenged by the discovery of *M. heureka*, which led to the hypothesis of Martialinae as the primary sister lineage to all other extant ants [16], though reanalyses of these data suggested Martialinae might instead be sister to a clade of poneroids and formicoids [46]. Subsequent genome-scale analyses using ultraconserved elements (UCEs) or whole-genome data have supported a combined Leptanillinae + Martialinae clade as sister to all other ants [11,47,48,49]. Recent works by Cai [50] and Tao et al. [17] instead recover Martialinae alone as sister to all ants excluding Leptanillinae.

In our principal concatenated ML analyses, Martialinae was recovered as the sister lineage to the remaining Formicidae. However, a closer examination of our results, particularly through Four-cluster Likelihood Mapping and partitioning sensitivity tests, highlights substantial phylogenetic discordance and model sensitivity at this node. When codon positions were partitioned separately, *M. heureka* was unexpectedly placed deep within Formicidae, albeit without statistical support, likely reflecting model instability or parameter over-parameterization. Furthermore, FcLM revealed distinct, competing signals among different dataset types: amino acid sequences favored a Leptanillinae + Martialinae clade or a Leptanillinae + Poneroid clade, whereas nucleotide datasets supported Leptanillinae as sister to the remaining poneroids.

Rather than providing a definitive resolution, our mitogenomic data illustrate how deep-node inference in ants can be confounded by systematic biases. Mitogenomes are prone to high substitution rates, nucleotide compositional heterogeneity, and saturation—particularly at third codon positions, as demonstrated by the substitution saturation analysis (*Iss > Iss*.*cAsym*). These evolutionary characteristics can induce long-branch attraction (LBA) or model misspecification. Furthermore, outgroup sensitivity remains a critical consideration when inferring the deepest nodes of the ant phylogeny. While selecting diverse outgroups is essential, long outgroup branches can exacerbate long-branch attraction (LBA) [51], potentially distorting the relationships among early-diverging ant lineages. In our initial phylogenetic reconstructions, several outgroup taxa (*Sclerodermus pupariae*, *Cephalonomia gallicola*, and *Vanhornia eucnemidarum*) exhibited exceptionally long branch lengths. However, sequential removal of these species did not alter the placement of the major basal ant lineages (see Supplementary Materials, Figures S10 and S11). These results indicate that the inferred deep relationships within Formicidae are robust to alternative outgroup selections.

Although removing heterogeneous taxa did not alter our primary ML topologies, the lingering conflict between nucleotide, amino acid, and FcLM signals indicates that mitochondrial sequences alone carry conflicting phylogenetic signals for the earliest ant divergences. Consequently, while our primary concatenated trees support Martialinae as the sister group to all other ants, this placement should be interpreted cautiously rather than as an unassailable resolution.

### 4.2. Intersubfamilial Relationships Within Formicidae

Beyond the primary root, our mitogenomic analyses successfully recovered the monophyly of Formicidae and all subfamilies represented by multiple species, reaffirming the broad utility of mitogenomes for lower-level phylogenetics. Previous studies often divided the family Formicidae into three major groups: the leptanilloid clade, a basal lineage comprising a single subfamily (Leptanillinae); the poneroid clade, which initially included five subfamilies (Agroecomyrmecinae, Amblyoponinae, Paraponerinae, Ponerinae, and Proceratiinae) [14,15] and was later expanded to include Apomyrminae [11,47,48,49]; and the formicoid clade, containing the remaining eight to nine subfamilies [11,47,48,49]. In the present study, the lineages corresponding to these three clades were all recovered as monophyletic. However, the internal relationships within each clade varied across datasets and analytical methods. Specifically, within the poneroid clade, inter-subfamilial relationships remain unstable. Nevertheless, a sister-group relationship between Apomyrminae and Amblyoponinae was supported by the amino acid dataset regardless of the inference method used, a finding consistent with previous studies using nuclear gene fragments [47], UCEs [48,49], and whole-genome datasets [16].

Within the formicoid clade, a sister-group relationship between Myrmeciinae and Pseudomyrmecinae was retrieved by the amino acid dataset, with four-cluster likelihood mapping consistently showing stronger support for this topology over alternative hypotheses. This relationship is also congruent with earlier studies [11,14,15,47,48,49]. Furthermore, two additional sister-group relationships, namely Aneuretinae + Dolichoderinae, and Heteroponerinae + Ectatomminae, were consistently recovered with strong nodal support across all analyses using our mitogenomic data. These clades align with findings from the previous literature [11,14,15,47,48,49].

Although recent taxonomic revisions have proposed subsuming Heteroponerinae within Ectatomminae [9], our dataset included only a single species of Heteroponerinae due to constraints on whole-genome data availability; thus, extended taxon sampling incorporating additional heteroponerine genera (*Acanthoponera* and *Aulacopone*) will be essential to test the subfamily status of Heteroponerinae thoroughly from a mitogenomic perspective.

Intriguingly, our mitogenomic data revealed two topological arrangements that diverge from the prevailing nuclear phylogenomic consensus. We consistently recovered a sister-group relationship between Myrmicinae and Formicinae. While this relationship was proposed in early molecular studies [14,15], recent large-scale UCE and phylogenomic works favor Formicinae + (Myrmicinae + Ectatomminae) [11,47,48,49]. Second, three of our four principal ML analyses positioned (Heteroponerinae + Ectatomminae) as sister to (Myrmicinae + Formicinae). In contrast, only the BI analysis of the amino acid dataset placed (Heteroponerinae + Ectatomminae) as sister to (Aneuretinae + Dolichoderinae), a placement that FcLM analysis also favored.

The discordances between mitogenomic and nuclear genomic topologies likely stem from fundamental differences in evolutionary dynamics. Organellar genomes reflect a single, non-recombining locus subject to distinct selection pressures, maternal inheritance, and pronounced compositional biases [52,53]. While mitogenomics provides critical complementary evidence and strong signal for many major clades, caution must be exercised when conflicting mitogenomic and nuclear signals arise. Resolving these persistent inter-subfamilial ambiguities will require broader taxon sampling, particularly for species-sparse and early-branching lineages, paired with specialized models designed to account for site- and lineage-specific compositional heterogeneity [47,50].

## 5. Conclusions

In this study, we significantly expanded the mitogenomic sampling of Formicidae by sequencing four new mitogenomes and assembling additional datasets from published resources. Our analyses robustly support the monophyly of Formicidae and all sampled multi-species subfamilies. Under the principal concatenated Maximum Likelihood analyses, *M. heureka* (Martialinae) was recovered as the sister lineage to all other extant ants. However, extensive sensitivity assessments, including likelihood mapping, alternative partitioning schemes, and Bayesian inference, revealed underlying signal conflict and dataset dependence regarding the exact placements of Martialinae and Leptanillinae. Excluding Martialinae, the remaining ants split into two major clades corresponding to the poneroid and formicoid lineages. Within these groups, our mitogenomic framework firmly validated several key sister-group relationships, such as Aneuretinae + Dolichoderinae, Heteroponerinae + Ectatomminae, and Apomyrminae + Amblyoponinae. Meanwhile, the recovery of a Myrmicinae + Formicinae clade highlights an intriguing divergence from nuclear phylogenomic trees. Overall, while mitogenomic data provide powerful resolution for most intersubfamilial relationships, deep basal nodes retain subtle signal conflicts driven by substitution saturation and compositional bias. Addressing these remaining deep-node uncertainties will necessitate balanced sampling across species-sparse lineages and the integration of nuclear genome-scale data under sophisticated heterogeneity-conscious analytical models.

## Data Availability

Annotated GenBank files for the newly sequenced mitogenomes are available in the Supplementary Materials. The core alignments, tree files, FcLM raw data files, and partition schemes, etc., used in this study can be available in Figshare (https://figshare.com/s/e7b860293677caa523c3, accessed on 3 September 2026; https://doi.org/10.6084/m9.figshare.33394234).

## Supplementary Materials

The following supporting information can be downloaded at: https://www.mdpi.com/article/10.3390/biology15181591/s1, Figure S1: Maximum Likelihood phylogenetic tree of ants inferred from the 227taxa_PCG_NT123 nucleotide dataset using IQ-TREE under the gene partition scheme. Numbers at nodes represent SH-aLRT support/UFBoot values (%). Figure S2: Maximum Likelihood phylogenetic tree of ants inferred from the 227taxa_PCG_NT12 nucleotide dataset using IQ-TREE under the gene partition scheme. Numbers at nodes represent SH-aLRT support/UFBoot values (%). Figure S3: Maximum Likelihood phylogenetic tree of ants inferred from the 227taxa_PCG_AA amino acid dataset using IQ-TREE under the gene partition scheme. Numbers at nodes represent SH-aLRT support/UFBoot values (%). Figure S4: Phylogenetic tree inferred from the PCG_AA dataset using PhyloBayes MPI under the site-heterogeneous CAT-GTR mixture model combined with the empirical mtART exchangeability matrix. Values at nodes represent Bayesian posterior probabilities. The scale bar indicates substitutions per site. Figure S5: Maximum Likelihood phylogenetic tree of ants inferred from the 223taxa_PCG_NT123 nucleotide dataset using IQ-TREE under the gene partition scheme. Numbers at nodes represent SH-aLRT support/UFBoot values (%). Figure S6: Maximum Likelihood phylogenetic tree of ants inferred from the 223taxa_PCG_NT12 nucleotide dataset using IQ-TREE under the gene partition scheme. Numbers at nodes represent SH-aLRT support/UFBoot values (%). Figure S7: Maximum Likelihood phylogenetic tree of ants inferred from the 223taxa_PCG_AA amino acid dataset using IQ-TREE under the gene partition scheme. Numbers at nodes represent SH-aLRT support/UFBoot values (%). Figure S8: Maximum Likelihood phylogenetic tree of ants inferred from the 227taxa_PCG_NT123 nucleotide dataset using IQ-TREE under the gene × codon-position partition scheme. Numbers at nodes represent SH-aLRT support/UFBoot values (%). Figure S9: Maximum Likelihood phylogenetic tree of ants inferred from the 227taxa_PCG_NT12 nucleotide dataset using IQ-TREE under the gene × codon-position partition scheme. Numbers at nodes represent SH-aLRT support/UFBoot values (%). Figure S10: Maximum Likelihood phylogenetic tree of ants inferred from the 225taxa_PCG_AA amino acid dataset (with two species of Bethylidae removed) using IQ-TREE under the gene partition scheme. Numbers at nodes represent SH-aLRT support/UFBoot values (%). Figure S11: Maximum Likelihood phylogenetic tree of ants inferred from the 224taxa_PCG_AA amino acid dataset (with two species of Bethylidae and the species *Vanhornia eucnemidarum* removed) using IQ-TREE under the gene partition scheme. Numbers at nodes represent SH-aLRT support/UFBoot values (%). Table S1: Summary statistics for each alignment. Table S2: Species analyzed in this study, including taxonomic classifications, GenBank accession numbers, and associated references [54,55,56,57,58,59,60,61,62,63,64,65,66,67,68,69,70,71,72,73,74,75,76]. Table S3: Summary of sampled taxa compared to extant valid genera and species richness. Table S4: Substitution saturation tests. Table S5: Model selection statistics and phylogenetic fit for gene-only versus gene × codon-position partitioning schemes evaluated in IQ-TREE. Supplementary Material: Sequences of the four newly sequenced mitogenomes in GenBank format.

## Abbreviations

The following abbreviations are used in this manuscript:

Mitogenome	Mitochondrial genome
PCGs	Protein-coding genes
tRNAs	Transfer RNAs
DNA	Deoxyribonucleic acid
BOLD	Barcode of Life Data System
ID%	Identity percentage
Bp	Base pair
ML	Maximum likelihood
BI	Bayesian inference
UFBoot	Ultrafast bootstrap
BPP	Bayesian posterior probabilities
FcLM	Four-cluster Likelihood Mapping
SH-aLRT	Shimodaira-Hasegawa-like approximate likelihood-ratio
ESS	Effective Sample Size
Iss	Index of Substitution Saturation
Iss.cSym	Critical Index of Substitution Saturation assuming Symmetric Tree
Iss.cAsym	Critical Index of Substitution Saturation assuming Asymmetrical Tree
N_OTU_	Number of Operational Taxonomic Units
AICc	Corrected Akaike information criterion
BIC	Bayesian information criterion
