# Supplementary material for: Insights into Ant Phylogeny from Mitogenomic Sampling Across All Extant Subfamilies
Source: Biology (Basel). 2026 Sep 9;15(18):1591. doi: 10.3390/biology15181591 (PMC13603798; doi:10.3390/biology15181591)
Supplement: Supplementary file 1 [file biology-15-01591-s001.zip › Supplementary_figures/Figure S3 227taxa_PCG_AA_gene_partition_ML.tre.pdf]

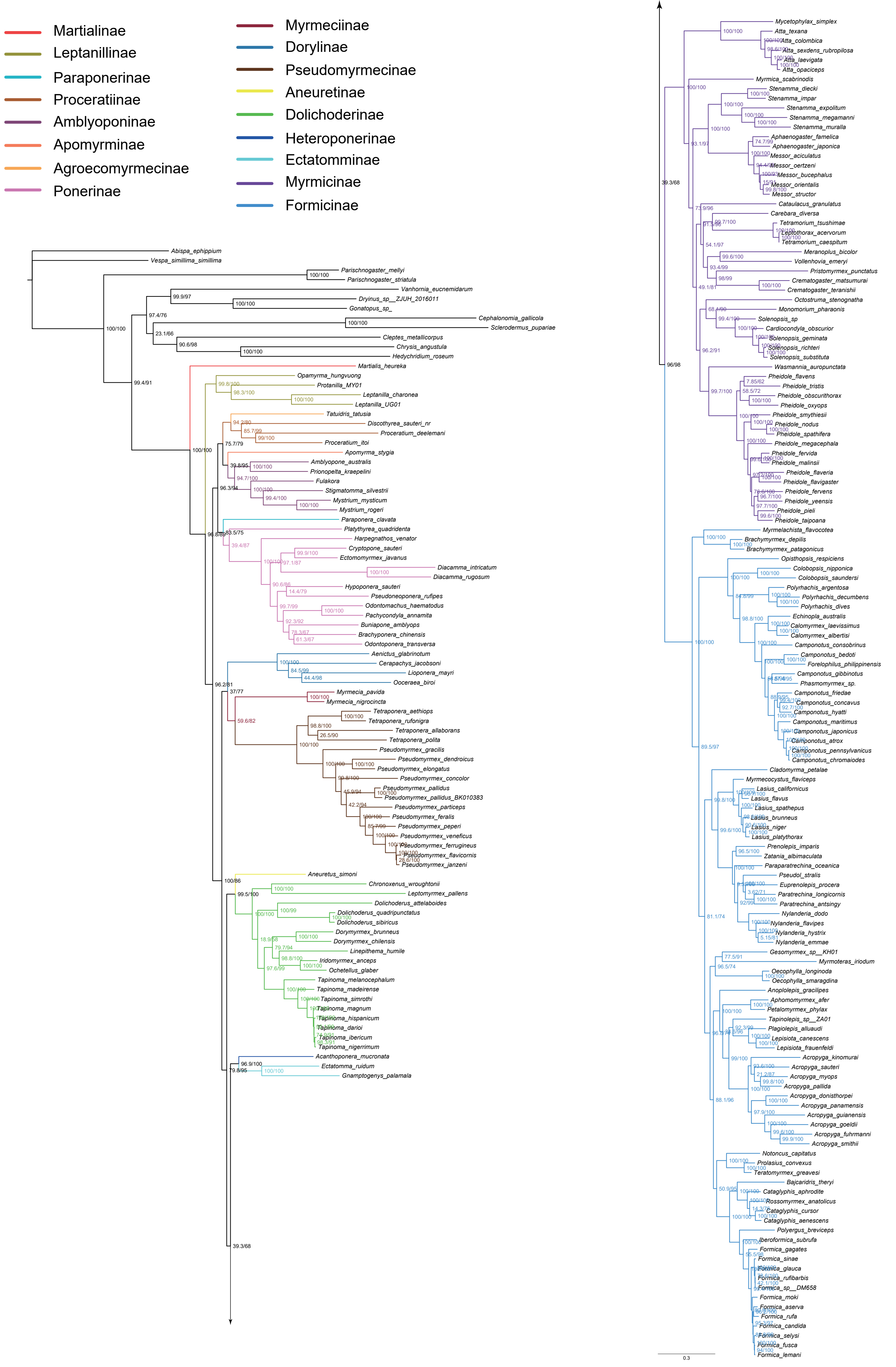

Martialinae
Leptanillinae
Paraponerinae
Proceratiinae
Amblyoponinae
Apomyrminae
Agroecomyrmecinae
Ponerinae
Myrmeciinae
Dorylinae
Pseudomyrmecinae
Aneuretinae
Dolichoderinae
Heteroponerinae
Ectatomminae
Myrmicinae
Formicinae
Abispa_ephippium
Vespa_simillima_simillima
Parischnogaster_mellyi
Parischnogaster_striatula
Vanhornia_eucnemidarum
Dryinus_sp__ZJUH_2016011
Gonatopus_sp_
Cephalonomia_gallicola
Sclerodermus_pupariae
Cleptes_metallicorpus
Chrysis_angustula
Hedychridium_roseum
Martialis_heureka
Opamyrma_hungvuong
Protanilla_MY01
Leptanilla_charonea
Leptanilla_UG01
Tatuidris_tatusia
Discothyrea_sauteri_nr
Proceratium_deelemani
Proceratium_itoi
Apomyrma_stygia
Amblyopone_australis
Prionopelta_kraepelini
Fulakora
Stigmatomma_silvestrii
Mystrium_mysticum
Mystrium_rogeri
Paraponera_clavata
Platythyrea_quadridenta
Harpegnathos_venator
Cryptopone_sauteri
Ectomomyrmex_javanus
Diacamma_intricatum
Diacamma_rugosum
Hypoponera_sauteri
Pseudoneoponera_rufipes
Odontomachus_haematodus
Pachycondyla_annamita
Buniapone_amblyops
Brachyponera_chinensis
Odontoponera_transversa
Aenictus_glabrinotum
Cerapachys_jacobsoni
Liponera_mayri
Ooceraea_biroi
Myrmecia_pavida
Myrmecia_nigrocincta
Tetraponera_aethiops
Tetraponera_rufonigra
Tetraponera_allaborans
Tetraponera_polita
Pseudomyrmex_gracilis
Pseudomyrmex_dendroicus
Pseudomyrmex_elongatus
Pseudomyrmex_concolor
Pseudomyrmex_pallidus
Pseudomyrmex_pallidus_BK010383
Pseudomyrmex_particeps
Pseudomyrmex_feralis
Pseudomyrmex_peperi
Pseudomyrmex_veneficus
Pseudomyrmex_ferrugineus
Pseudomyrmex_flavicornis
Pseudomyrmex_janzeni
Aneuretus_simoni
Chronoxenus_wroughtonii
Leptomyrmex_pallens
Dolichoderus_attelaboides
Dolichoderus_quadripunctatus
Dolichoderus_sibiricus
Dorymyrmex_brunneus
Dorymyrmex_chilensis
Linepithema_humile
Iridomyrmex_anceps
Ochetellus_glaber
Tapinoma_melanocephalum
Tapinoma_madeirense
Tapinoma_simrothi
Tapinoma_magnum
Tapinoma_hispanicum
Tapinoma_darioi
Tapinoma_ibericum
Tapinoma_nigerrimum
Acanthoponera_mucronata
Ectatomma_ruidum
Gnamptogenys_palamala
Mycetophylax_simplex
Atta_texana
Atta_colombica
Atta_sexdens_rubropilosa
Atta_laevigata
Atta_opaciceps
Myrmica_scabrinodis
Stenamma_diecki
Stenamma_impar
Stenamma_expolitum
Stenamma_megamanni
Stenamma_muralla
Aphaenogaster_famelica
Aphaenogaster_japonica
Messor_aciculatus
Messor_oertzeni
Messor_bucephalus
Messor_orientalis
Messor_structor
Cataulacus_granulatus
Carebara_diversa
Tetramorium_tsushimae
Leptothorax_acervorum
Tetramorium_caespitum
Meranoplus_bicolor
Vollenhovia_emeryi
Pristomyrmex_punctatus
Crematogaster_matsumurai
Crematogaster_teranishii
Octostruma_stenognatha
Monomorium_pharaonis
Solenopsis_sp
Cardiocondyla_obscurior
Solenopsis_geminata
Solenopsis_richteri
Solenopsis_substituta
Wasmannia_auropunctata
Pheidole_flavens
Pheidole_tristis
Pheidole_obscurithorax
Pheidole_oxyops
Pheidole_smythiesii
Pheidole_nodus
Pheidole_spathifera
Pheidole_megacephala
Pheidole_fervida
Pheidole_malinsii
Pheidole_flaveria
Pheidole_flavigaster
Pheidole_fervens
Pheidole_yeensis
Pheidole_pieli
Pheidole_taipoana
Myrmelachista_flavocotea
Brachymyrmex_depilis
Brachymyrmex_patagonicus
Opisthopsis_respiciens
Colobopsis_nipponica
Colobopsis_saundersi
Polyrhachis_argentosa
Polyrhachis_decumbens
Polyrhachis_dives
Echinopla_australis
Calomyrmex_laevissimus
Calomyrmex_albertisi
Camponotus_consobrinus
Camponotus_bedoti
Forelophilus_philippinensis
Camponotus_gibbinotus
Phasmomyrmex_sp.
Camponotus_friedae
Camponotus_concavus
Camponotus_hyatti
Camponotus_maritimus
Camponotus_japonicus
Camponotus_atrox
Camponotus_pennsylvanicus
Camponotus_chromaiodes
Cladomyrma_petalae
Myrmecocystus_flaviceps
Lasius_californicus
Lasius_flavus
Lasius_spathepus
Lasius_brunneus
Lasius_niger
Lasius_platythorax
Prenolepis_imparis
Zatania_albimaculata
Paraparatrechina_oceanica
Pseudolasius_stralis
Euprenolepis_procera
Paratrechina_longicornis
Paratrechina_antsingy
Nylanderia_dodo
Nylanderia_flavipes
Nylanderia_hystrix
Nylanderia_emmae
Gesomyrmex_sp__KH01
Myrmoteras_iriodum
Oecophylla_longinoda
Oecophylla_smaragdina
Anoplolepis_gracilipes
Aphomomyrmex_afer
Petalomyrmex_phylax
Tapinolepis_sp__ZA01
Plagiolepis_alluaudi
Lepisiota_canescens
Lepisiota_frauenfeldi
Acropyga_kinomurai
Acropyga_sauteri
Acropyga_myops
Acropyga_pallida
Acropyga_donisthorpei
Acropyga_panamensis
Acropyga_guianensis
Acropyga_goeldii
Acropyga_fuhrmanni
Acropyga_smithii
Notoncus_capitatus
Prolasius_convexus
Teratomyrmex_greavesi
Bajcaridris_theryi
Cataglyphis_aphrodite
Rossomyrmex_anatolicus
Cataglyphis_cursor
Cataglyphis_aenescens
Polyergus_breviceps
Iberoformica_subrufa
Formica_gagates
Formica_sinae
Formica_glauca
Formica_rufibarbis
Formica_sp__DM658
Formica_moki
Formica_aserva
Formica_rufa
Formica_candida
Formica_selysi
Formica_fusca
Formica_lemani
0.3
