# Supplementary material for: Insights into Ant Phylogeny from Mitogenomic Sampling Across All Extant Subfamilies
Source: Biology (Basel). 2026 Sep 9;15(18):1591. doi: 10.3390/biology15181591 (PMC13603798; doi:10.3390/biology15181591)
Supplement: Supplementary file 1 [file biology-15-01591-s001.zip › Supplementary_figures/Figure S4 PCG_AA_PhyloBayes_tree.pdf]

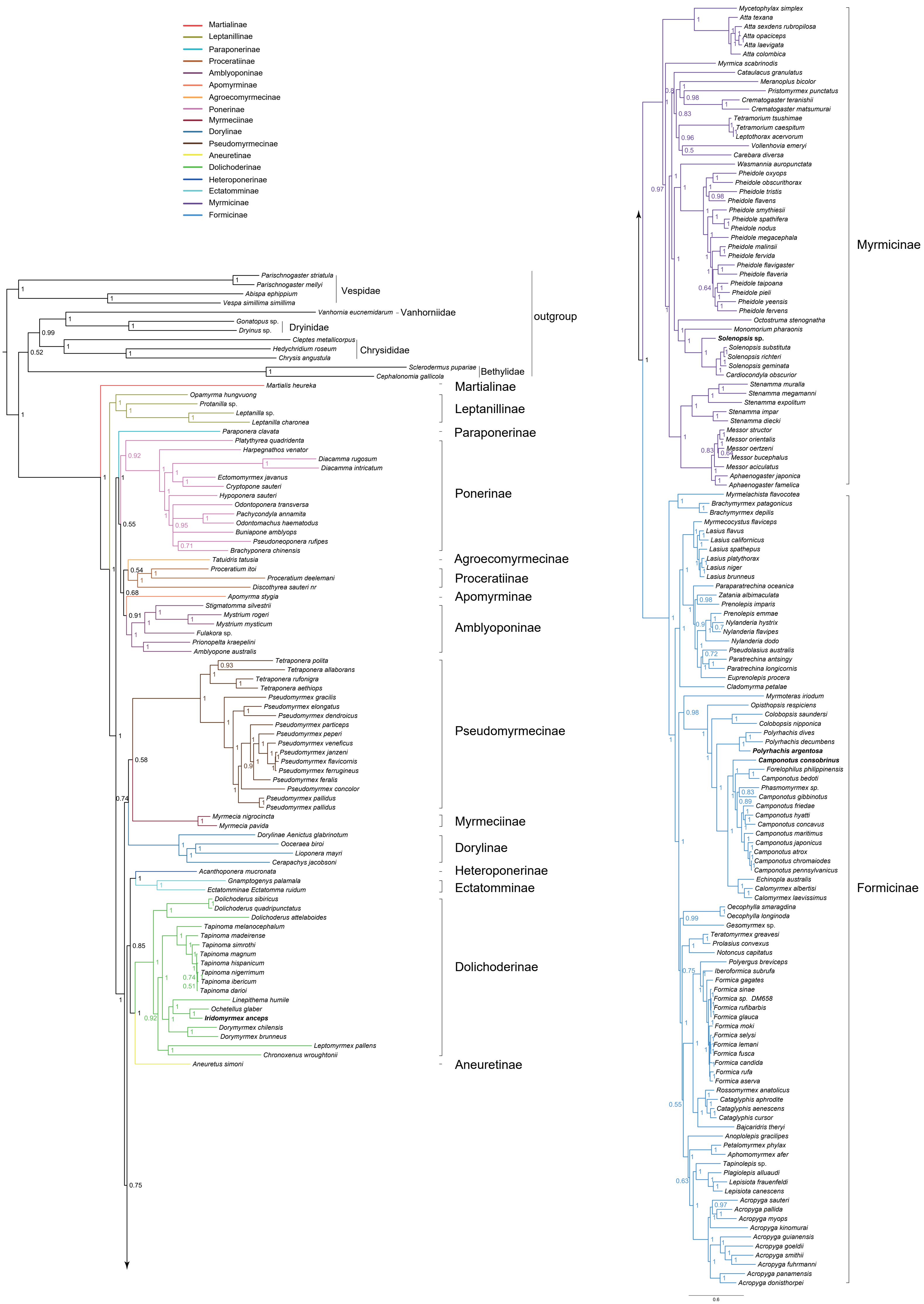
Martialinae
Leptanillinae
Paraponerinae
Proceratiinae
Amblyoponinae
Apomyrminae
Agroecomyrmecinae
Ponerinae
Myrmeciinae
Dorylinae
Pseudomyrmecinae
Aneuretinae
Dolichoderinae
Heteroponerinae
Ectatomminae
Myrmicinae
Formicinae
Parischnogaster striatula
Parischnogaster mellyi
Abispa ephippium
Vespa simillima simillima
Vespidae
Vanhornia eucnemidarum
Vanhorniidae
Gonatopus sp.
Dryinus sp.
Dryinidae
Cleptes metallicorpus
Hedychridium roseum
Chrysis angustula
Chrysididae
Sclerodermus pupariae
Cephalonomia gallicola
Bethylidae
outgroup
Martialis heureka
Opamyrma hungvuong
Protanilla sp.
Leptanilla sp.
Leptanilla charonea
Paraponera clavata
Platythyrea quadridenta
Harpegnathos venator
Diacamma rugosum
Diacamma intricatum
Ectomomyrmex javanus
Cryptopone sauteri
Hypoponera sauteri
Odontoponera transversa
Pachycondyla annamita
Odontomachus haematodus
Buniapone amblyops
Pseudoneoponera rufipes
Brachyponera chinensis
Tatuidris tatusia
Proceratium itoi
Proceratium deelemani
Discothyrea sauteri nr
Apomyrma stygia
Stigmatomma silvestrii
Mystrium rogeri
Mystrium mysticum
Fulakora sp.
Prionopelta kraepelini
Amblyopone australis
Tetraponera polita
Tetraponera allaborans
Tetraponera rufonigra
Tetraponera aethiops
Pseudomyrmex gracilis
Pseudomyrmex elongatus
Pseudomyrmex dendroicus
Pseudomyrmex particeps
Pseudomyrmex peperi
Pseudomyrmex veneficus
Pseudomyrmex janzeni
Pseudomyrmex flavicornis
Pseudomyrmex ferrugineus
Pseudomyrmex feralis
Pseudomyrmex concolor
Pseudomyrmex pallidus
Pseudomyrmex pallidus
Myrmecia nigrocincta
Myrmecia pavida
Dorylinae Aenictus glabrinotum
Ooceraea biroi
Lioponera mayri
Cerapachys jacobsoni
Acanthoponera mucronata
Gnamptogenys palamala
Ectatomminae Ectatomma ruidum
Dolichoderus sibiricus
Dolichoderus quadripunctatus
Dolichoderus attelaboides
Tapinoma melanocephalum
Tapinoma madeirense
Tapinoma simrothi
Tapinoma magnum
Tapinoma hispanicum
Tapinoma nigerrimum
Tapinoma ibericum
Tapinoma darioi
Linepithema humile
Ochetellus glaber
Iridomyrmex anceps
Dorymyrmex chilensis
Dorymyrmex brunneus
Leptomyrmex pallens
Chronoxenus wroughtonii
Aneuretus simoni
Mycetophylax simplex
Atta texana
Atta sexdens rubropilosa
Atta opaciceps
Atta laevigata
Atta colombica
Myrmica scabrinodis
Cataulacus granulatus
Meranoplus bicolor
Pristomyrmex punctatus
Crematogaster teranishii
Crematogaster matsumurai
Tetramorium tsushimae
Tetramorium caespitum
Leptothorax acervorum
Vollenhovia emeryi
Carebara diversa
Wasmannia auropunctata
Pheidole oxyops
Pheidole obscurithorax
Pheidole tristis
Pheidole flavens
Pheidole smythiesii
Pheidole spathifera
Pheidole nodus
Pheidole megacephala
Pheidole malinsii
Pheidole fervida
Pheidole flavigaster
Pheidole flaveria
Pheidole taipoana
Pheidole pieli
Pheidole yeensis
Pheidole fervens
Octostruma stenognatha
Monomorium pharaonis
Solenopsis sp.
Solenopsis substituta
Solenopsis richteri
Solenopsis geminata
Cardiocondyla obscurior
Stenamma muralla
Stenamma megamanni
Stenamma expolitum
Stenamma impar
Stenamma diecki
Messor structor
Messor orientalis
Messor oertzeni
Messor bucephalus
Messor aciculatus
Aphaenogaster japonica
Aphaenogaster famelica
Myrmelachista flavocotea
Brachymyrmex patagonicus
Brachymyrmex depilis
Myrmecocystus flaviceps
Lasius flavus
Lasius californicus
Lasius spathepus
Lasius platythorax
Lasius niger
Lasius brunneus
Paraparatrechina oceanica
Zatania albimaculata
Prenolepis imparis
Prenolepis emmae
Nylanderia hystrix
Nylanderia flavipes
Nylanderia dodo
Pseudolasius australis
Paratrechina antsingy
Paratrechina longicornis
Euprenolepis procera
Cladomyrma petalae
Myrmoteras iriodum
Opisthopsis respiciens
Colobopsis saundersi
Colobopsis nipponica
Polyrhachis dives
Polyrhachis decumbens
Polyrhachis argentosa
Camponotus consobrinus
Forelophilus philippinensis
Camponotus bedoti
Phasmomyrmex sp.
Camponotus gibbinotus
Camponotus friedae
Camponotus hyatti
Camponotus concavus
Camponotus maritimus
Camponotus japonicus
Camponotus atrox
Camponotus chromaiodes
Camponotus pennsylvanicus
Echinopla australis
Calomyrmex albertisi
Calomyrmex laevissimus
Oecophylla smaragdina
Oecophylla longinoda
Gesomyrmex sp.
Teratomyrmex greavesi
Prolasius convexus
Notoncus capitatus
Polyergus breviceps
Iberoformica subrufa
Formica gagates
Formica sinae
Formica sp. DM658
Formica rufibarbis
Formica glauca
Formica moki
Formica selysi
Formica lemani
Formica fusca
Formica candida
Formica rufa
Formica aserva
Rossomyrmex anatolicus
Cataglyphis aphrodite
Cataglyphis aenescens
Cataglyphis cursor
Bajcaridris theryi
Anoplolepis gracilipes
Petalomyrmex phylax
Aphomomyrmex afer
Tapinolepis sp.
Plagiolepis alluaudi
Lepisiota frauenfeldi
Lepisiota canescens
Acropyga sauteri
Acropyga pallida
Acropyga myops
Acropyga kinomurai
Acropyga guianensis
Acropyga goeldii
Acropyga smithii
Acropyga fuhrmanni
Acropyga panamensis
Acropyga donisthorpei
0.6
